# Comprehensive Mitochondrial Metabolic Shift during the Critical Node of Seed Ageing in Rice

**DOI:** 10.1371/journal.pone.0148013

**Published:** 2016-04-28

**Authors:** Guangkun Yin, James Whelan, Shuhua Wu, Jing Zhou, Baoyin Chen, Xiaoling Chen, Jinmei Zhang, Juanjuan He, Xia Xin, Xinxiong Lu

**Affiliations:** 1 National Genebank, Institute of Crop Science, Chinese Academy of Agricultural Sciences, Beijing 100081, China; 2 Australian Research Council Centre of Excellence in Plant Energy Biology, School of Life Science, La Trobe University, Bundoora, Victoria 3083, Australia; 3 Key Laboratory of Ministry of Education for Genetics, Breeding and Multiple Utilization of Crops, Fujian Agriculture & Forestry University, Fuzhou, Fujian, 350002, China; Universidade Federal de Vicosa, BRAZIL

## Abstract

The critical node (CN) in seed aging in rice (*Oryza sativa*) is the transformation from Phase I (P-I) to Phase II (P-II) of the reverse S-shaped curve (RS-SC). Although mitochondrial dysfunction plays a key role in seed ageing, the metabolic shift in the CN remains poorly understood. Here, we investigated the mitochondrial regulatory mechanisms during the CN of rice seed ageing. We showed that during the CN of seed ageing, the mitochondrial ultrastructure was impaired, causing oxygen consumption to decrease, along with cytochrome *c* (cyt *c*) oxidase and malate dehydrogenase (MDH) activity. In addition, the transcript levels for the alternative pathway of the electron transport chain (ETC) were significantly induced, whereas the transcripts of the cytochrome oxidase (COX) pathway were inhibited. These changes were concomitant with the down-regulation of mitochondrial protein levels related to carbon and nitrogen metabolism, ATP synthase (ATPase) complex, tricarboxylic acid cycle (TCA) cycle, mitochondrial oxidative enzymes, and a variety of other proteins. Therefore, while these responses inhibit the production of ATP and its intermediates, signals from mitochondria (such as the decrease of cyt *c* and accumulation of reactive oxygen species (ROS)) may also induce oxidative damage. These events provide considerable information about the mitochondrial metabolic shifts involved in the progression of seed ageing in the CN.

## Introduction

For the conservation of plant resources, more than 7,400,000 accessions have been collected and preserved worldwide [1]. The National Genebank of China currently holds around 395,000 accessions at -18°C; these accessions represent the base collection of commercial crops and their corresponding wild relatives including rice. Incidentally, seeds continue to slowly age under the conventional storing conditions of low temperature and low moisture [2, 3]. A notable characteristic of seed viability during ageing is the reverse S-shaped curve (RS-SC) which comprises of three phases, the plateau phase (Phase I, P-I) followed by the rapid decreasing phase (Phase II, P-II) and to complete, the slow decrease phase (Phase III, P-III), however, it is the transformation from P-I to P-II, also known as the critical node (CN) that is extremely important for seed conservation. Close monitoring of 14,000 seed accessions over a 20-year storage period showed that 1.1% of the seed accessions decreased to below 70% seed viability when entering P-II of RS-SC [4]. Therefore, as appropriate seed storage is essential for species conservation and seeds entering the CN towards P-II of RS-SC during storage can influence seed viability, understanding the mechanisms of seed ageing at the CN of RS-SC will improve our understanding of seed longevity during long term storage.

During the seedling stage, mitochondrial metabolism supplies energy compounds by converting intermediates into adenosine triphosphate (ATP) for cellular biosynthesis [5, 6]. Although dry mature seeds have poorly differentiated mitochondria, they still contain sufficient tricarboxylic acid (TCA) cycle enzymes and terminal oxidases to provide adequate ATP for energy support following imbibition [7]. As well as ATP, mitochondria also generate harmful reactive oxygen species (ROS) as a by-product of ATP production via the ETC (ETC) site [8]. Following RS-SC, age-related changes in mitochondrial function lead to accumulation of ROS; causing organ dysfunction and membrane system disorders [9, 10] as well as oxidative damage of mitochondrial proteins, DNA and lipids [11–13]. According to the free radical theory of ageing, the generation and quantity of ROS are crucial for the progression of seed ageing and other ageing-associated disorders [14, 15]. Additionally, changes in cellular ROS levels, energy production or redox status by mitochondrial function or dysfunction triggers various responses that regulate mitochondrial and nuclear gene expression pivotal to seed aging [16–21]. Therefore, as mitochondrial biogenesis is associated with seed aging, mitochondrial metabolism regulates ROS generation and ROS production initiates gene expression for seed ageing, it is clear that mitochondrial mechanisms regulate seed ageing.

Mitochondrial and nuclear encoded components of the TCA cycle and ETC orchestrate the development of unstructured promitochondria to typical cristae-rich mitochondria [22–25]. Expression of these TCA cycle and ETC components were altered in aged maize seeds [26]. In aged soybean seeds, mitochondrial activity significantly decrease, especially mitochondrial ascorbic acid and glutathione cycle activity, resulting in elevated ROS accumulation [27]. During controlled deterioration in elm (*Ulmus pumila* L.) seeds, mitochondrial dysfunction of cytochrome *c* (cyt *c*) initiates ROS production and programmed cell death [28]. Mitochondrial β-mercaptopyruvate sulfurtransferase was down regulated in aged Arabidopsis seeds [29]. While most descriptions of mitochondrial regulatory mechanisms during seed aging remain limited to the characterization of protein and gene expression, no direct studies have been carried out to investigate mitochondrial metabolic shifts at the CN of seed viability. Therefore, to better understand mitochondrial function at the CN towards P-II of RS-SC, the role of mitochondria in seed ageing must be defined by studying the earliest events of seed ageing.

Here, we use aged rice seeds at the CN development stage as material to elucidate mitochondrial regulatory mechanisms. Mitochondria were isolated in high purity from rice embryos after 48 h imbibition using Percoll density gradient centrifugation. We investigated mitochondrial morphology and activity, combined with the gene expression of components from the ETC. In addition, the cumulative changes of mitochondrial proteins were also analyzed using antibodies and mass spectrometry. The results provide novel insights into the regulatory effect of mitochondria on seed ageing, which can facilitate the comprehensive conservation of germplasm resources.

## Materials and Methods

### Plant material and treatment

Rice (*Oryza sativa* L. *japonica*. *nipponbare*) seeds were obtained from the Jiangxi Academy of Agricultural Sciences with germination rates and moisture contents of 99% and 12.3%, respectively. Seeds were artificially aged at 40°C and 75% relative humidity (RH) for various periods of time (3, 4, 7, 10 and 14 d). For germination analysis, seeds were germinated at 28°C in the dark [30] while rice embryos were collected from grains 48 h after imbibition.

### Electron microscopy

Following dissection to 1 mm^3^, rice embryos were fixed with 2.5% (v/v) glutaraldehyde in 50 mM sodium phosphate buffer (pH 7.2). Each sample was dehydrated by graded ethanol solution and the samples were embedded in spurr resin. Ultra-thin sections were stained with uranyl acetate followed by lead citrate and the processed sections were observed under an H-7500 transmission electron microscope (Hitachi, Japan).

### Mitochondrial isolation

Rice mitochondria were isolated from 48 h imbibed embryos using a modified protocol according to [22]. Following dissection, embryos (1200–1500) were quickly homogenized in grinding buffer (0.3 M mannitol, 2 mM EGTA, 0.5% (w/v) PVP-40, 0.5% (w/v) BSA, 20 mM cysteine, pH 7.5). All steps were carried out on ice or at 4°C. The homogenate was centrifuged at 2, 000 × g for 15 min, and the supernatant was collected. The pellet was re-suspended in grinding buffer and centrifuged again at 2, 000 × g for 15 min, and then the supernatant was collected again. The pooled supernatants were centrifuged at 12, 000 × g for 15 min. The pellet was re-suspended in wash buffer (0.3 M sucrose, 10 mM TES, pH 7.5) and centrifuged again at 2, 000 × g for 15 min. The supernatant was collected and centrifuged at 12, 000 × g for 15 min. This pellet was re-suspended again in wash buffer, which was crude mitochondria, and layered on top of 25% and 40% Percoll gradient in wash buffer. After centrifugation for 1 h at 40, 000 × g, the visible band at the 25–40% Percoll interface was collected, diluted with wash buffer, and centrifuged at 15, 000 × g for 15 min. The pellet was collected with 200 μL wash buffer which was highly purified mitochondria.

### Respiratory measurements and enzymes activity

O_2_ uptake was assayed using an O_2_ electrode (Hansatech, UK) in 1 mL reaction buffer containing 0.3 M sucrose, 10 mM TES-KOH (pH 7.5), 5 mM KH_2_PO_4_, 10 mM NaCl, 2 mM MgSO_4_ and 0.1% (w/v) BSA as well as NADH (1 mm), succinate (10 mm) and ADP (1 mm) according to Logan et al [22].

Enzyme activity was determined in isolated mitochondria. Cytochrome c oxidase (COX) activity was measured via cytochrome c oxidation during the absorbance decease at 550 nm and 25°C in the presence of 0.05% (w/v) Triton X-100 [31]. Mitochondrial malate dehydrogenase (MDH) activity was determined by monitoring the increase in absorbance at 340 nm and 25°C due to NADH production in the presence of 0.05% (w/v) Triton X-100 [32].

### Two dimensional and image analysis

Mitochondria protein extraction was achieved according to the Tris-phenol protocol. Mitochondrial protein was dissolved in sample buffer containing 7 M urea, 2 M thiourea, 2% CHAPS, 65 mM DTT, 0.2% pharmalyte (pH 5–8) according to Yin et al [33]. Approximately 500 μg in 300 uL sample buffer were applied to rehydrate gel strips with an immobilized linear pH gradient from 5 to 8 (ReadyStrip IPG Strip, 170 mm, pH 5–8, BioRad, USA). The protein was determined by the method of Bradford [34]. The first-dimensional IEF was performed at 20°C on a flat-bed electrophoresis unit (PROTEAN IEF Cell, BioRad, USA), as following: rehydration 12 h, 0 to 150 V in 15 min, from 150 V to 1,000 V in 1h, from 1,000 V to 8,000 V in 5 h, 8,000 V until a total of 60 kVh. The strip was then incubated for 15 min in each of the following solutions: (1) 6 M urea, 20% (v/v) glycerol, 2% (w/v) SDS, 375 mM Tris-HCl, pH 8.8, 2% (w/v) DTT; (2) 6 M urea, 20% (v/v) glycerol, 2% (w/v) SDS, 375 mM Tris-HCl, pH 8.8, 2.5% iodoacetamide. SDS-PAGE in the second dimension was conducted using homogenous 12% polyacrylamide gels with 4% stacking gels (Protean II, BioRad, USA). The running buffer contained 25 mM Tris (pH 8.3), 195 mM glycine, and 0.1% (w/v) SDS. Gel electrophoresis was performed at 250 V with circulating cooling. The gels were stained using 40% (v/v) methanol, 10% (v/v) acetic acid and 0.2% (w/v) CBB G250 and destained using 40% (v/v) methanol and 10% (v/v) acetic acid.

Gel images were obtained by a using a flatbed scanner (Umax, Taiwan), and analyzed by Image Master 2D Elite software (Amersham Biosciences, USA). Spot intensity was calculated according to the relative expression volume. Spots which changed > 2-fold were excised for protein identification.

### In-gel digestion, mass spectrometry and database searching

Protein spots were cut into 1 mm^3^ pieces. Gel slices were destained with 25 mM NH_4_CO_3_/50% acetonitrile until the CBB was removed. After dehydration by adding acetonitrile, gel pieces were digested for 15 min on ice in 25 μl of trypsin solution containing 0.1 mM trypsin in 25 mM NH_4_CO_3_. Excess trypsin solution was then removed. The solution (20 μl 25 mM NH_4_CO_3_) was added to overlay the gel pieces at 37°C for 12 h. The peptides solution was desalted with Zip-Tip C_18_ (Millipore, USA), which were eluted from the column in 2 μl of 0.1% TFA in 50% acetonitrile.

For matrix assisted laser desorption-ionization time of flight (MALDI-TOF)/TOF MS/MS analysis, 1μl of peptides solution and 1μl of matrix solution (1 mg·ml^–1^, a-cyano-4-hydroxycinnamic acid in 70% acetonitrile containing 0.1% TFA) was spotted onto the AnchorChip MALDI target plate (Bruker Daltonics, USA). Mass spectra were carried out on a MALDI-TOF/TOF mass spectrometer (Bruker Daltonics, USA) according to the method described by manual instruction.

MS data were analysed using the Mascot server by uploading with Biotools software (Bruker Daltonics, USA) and were searched against NCBInr protein database (version, 20130501, 25010123 sequences; 8625376125 residues). Search parameters were set as following: rice; proteolytic enzyme, trypsin; max missed cleavages, 1; fix modifications, carbamidomethyl (C); variable modifications, oxidation (M); peptide mass tolerance, 100 ppm; fragment mass tolerance, 0.5 Da. The Mowse score was evaluated the data obtained. To confirm protein identification, the best match is the one with the highest score which was at least more than 65. iPSORT (http://ipsort.hgc.jp/)was used to predict the subcellular location of the identified proteins.

### Immunoblot Analysis

Equal amounts of mitochondria protein (10 μg per lane) were loaded onto SDS-PAGE gels, transferred to PVDF or nitrocellulose membranes, blocked, and incubated with the following antibodies: AtpB, SHMT, IDH, VADC1, Cyt *c*, MnSOD, CAT, APX and GR (Agrisera, Sweden). A secondary antibody was used the anti-rabbit IgG (Agrisera, Sweden). Immunodetection was performed using the Chemiluminescent Substrate Kit (KPL, USA).

### Quantitative RT–PCR

Total RNA was isolated from 48 h imbibed embryos using the RNAprep pure plant kit (Tiangen, China) and DNase treated using the Tiangen on-column DNase digestion. cDNA synthesis were carried out using the FastQuant RT kit with gDNase (Tiangen, China). Quantitative RT-PCR was performed using the ABI 7900 fast Real Time PCR (Applied Biosystems, USA), and the Tiangen SuperReal PreMix Plus (SYBR Green) was used to quantify the differential expression and 20 μl of the reaction mixture was added to each well. The thermal cycling program was 95°C for 15 min, 40 cycles at 95°C for 15 s and 30 s at 60°C. Specific primer pairs (S1 Table) targeting selected genes were designed using Primer 5 software. Transcript profiles representing the relative message abundance were normalized to the unaged rice embryo.

### Statistical analysis

All data were analyzed as a one-variable general linear model procedure (analysis of variance) by SPSS (SPSS Inc., Chicago, IL, USA). Mean separations were performed with the least significant difference test and differences at *p*<0.05 were considered to be significant. Results presented were pooled across repeated experiments.

## Results

### Mitochondrial morphology during the critical node of seed ageing

**Fig 1:**
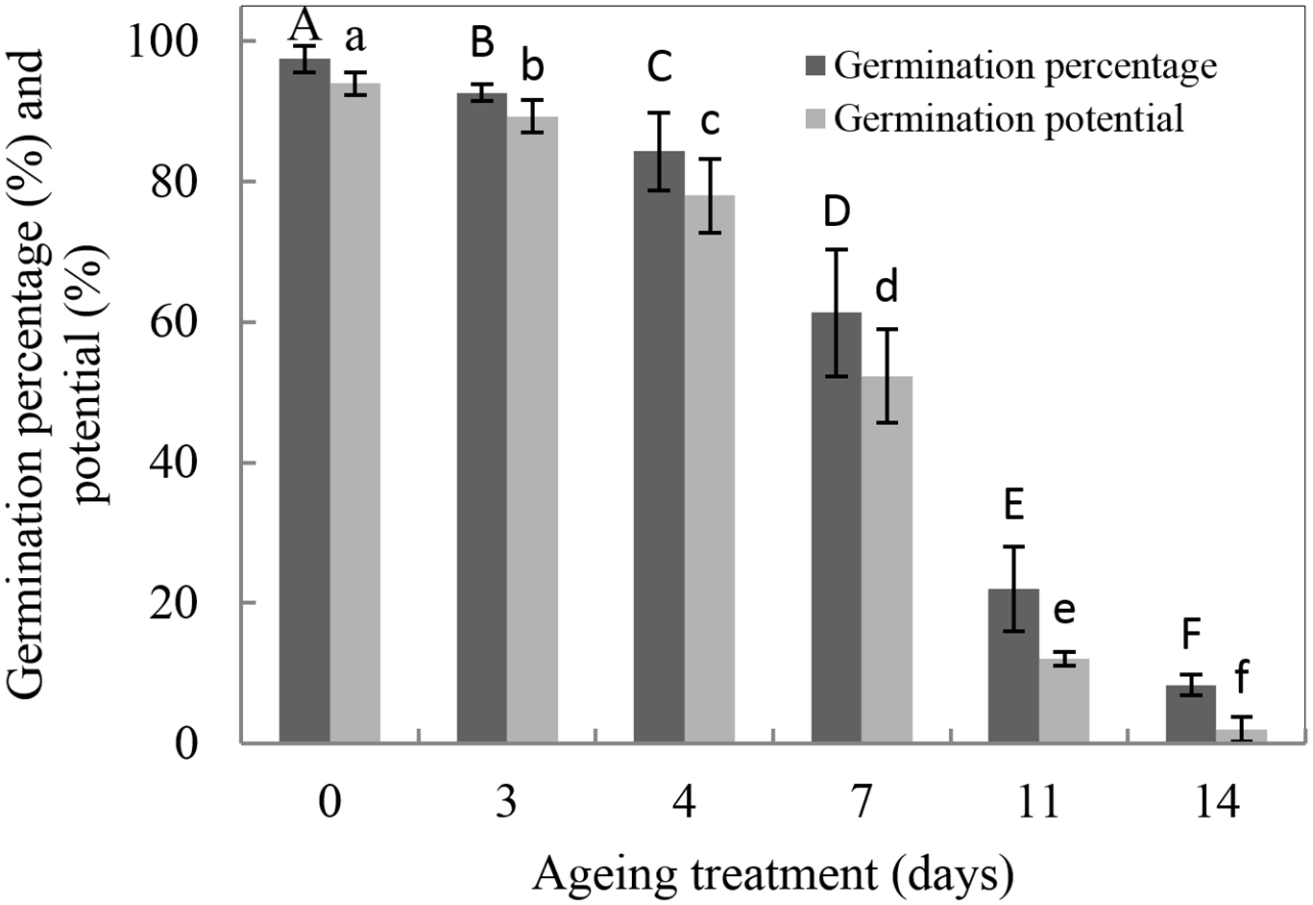
Influence of the artificial ageing treatment on rice germination percentage and potential. Data represent the mean ± standard deviation of three independent experiments. The results of all treatments differed significantly from the control (***p***< 0.05, one-way ANOVA, n = 3).

The CN of seed ageing is described in detail in our previous studies [3, 4], in brief, it is the stage at which the seed ageing curve transforms from P-I to P-II. As suggested by Lu et al[3],the P-I of rice seeds is when the germination percentage exceeds 88%. To acquire aged rice seeds in the CN, rice seeds were subject to an artificially aging treatment for 3 d, 4 d, 7 d, 11 d and 14 d at 40°C and 75% RH. The germination percentage significantly dropped from 97% to 92%, 84%, 61%, 22% and 8%, respectively (Fig 1), while the germination potential was significantly reduced from 94% to 89%, 78%, 52%, 12% and 2%, respectively (Fig 1), so we chose the seed viability at 4 d aging as the CN.

**Fig 2:**
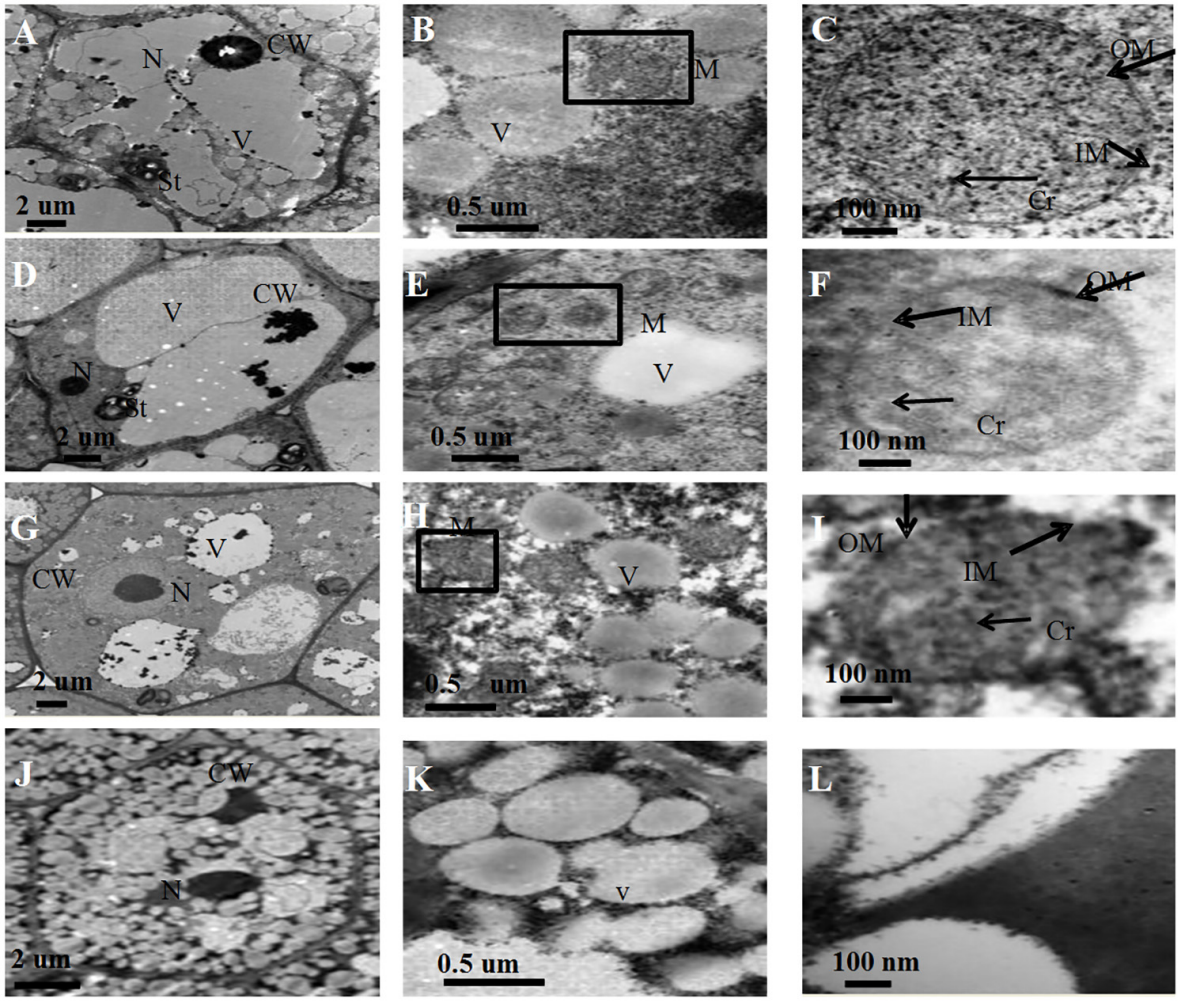
Transmission electron micrographs of rice embryos aged 0 d (A–C), 3 d (D–F), 4 d (G–I), and 7 d (J–L). N, nucleus; CW, cell wall; St, starch granule; V, vacuole; M, mitochondria; Cr, cristae; OM, outer membrane; IM, inner boundary membrane.

A morphological examination of rice embryos from the aged seeds after 48 h imbibition was performed using transmission electron microscopy. Numerous mature mitochondria with typical structures of well-developed cristae, an easily distinguishable outer and inner boundary membrane were observed in un-aged seed embryos (Fig 2C). The inner membrane and cristae decreased slightly in 3 d aged seed embryos (Fig 2F). In contrast, after 4 d ageing, little discernible internal structure remained (Fig 2I). After 7 d ageing, it was difficult to find mitochondria in the embryo (Fig 2L). In addition, with prolonged ageing treatment, the ultrastructure of the embryo cell was significantly altered after 48 h imbibition (Fig 2A, 2D, 2G and 2J). These results indicate that the integrity of the mitochondrial ultrastructure was seriously inhibited in rice embryos after 48 h imbibition in the CN.

### Transcript levels of the mitochondrial electron transport chain

**Fig 3:**
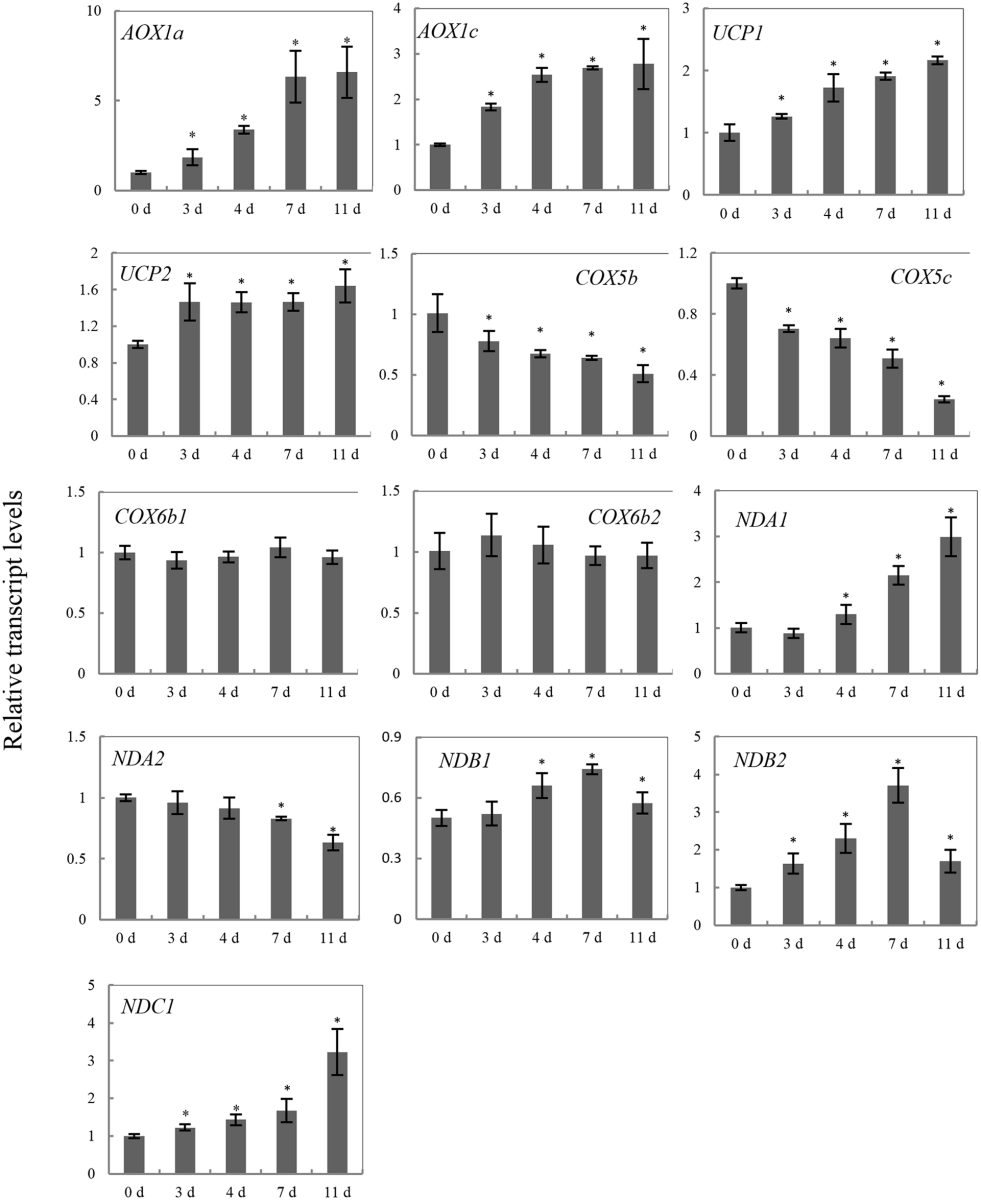
Abundance of the transcripts of mitochondrial components in rice embryos during germination. For each of the 13 transcripts investigated, a value of 1.0 was assigned to 0 d aged seed after imbibition 48 h and message abundance at the 3 d, 4 d, 7 d and 11 d aged seed was calculated relative to it. Data represent the mean ± standard deviation of 3 independent experiments. Asterisks indicate significant differences to 0 d aged seed (Student’s t-test; *p< 0.05).

Plant mitochondria have a branched ETC, which is essential for ATP production and contains alternative NADH dehydrogenase (ND), uncoupled protein (UCP), alternative oxidase (AOX) and cytochrome oxidase (COX) [35]. ND, AOX and UCP represent the non-phosphorylating ETC, which may be induced under stress to prevent ROS production [36–38]. To investigate the effects of limiting the ETC in aged rice seeds, 13 genes encoding the mitochondrial ETC were analyzed by quantitative RT-PCR (qRT-PCR) (S1 Table). The transcript abundance of these genes were examined in cDNA prepared from the 0 d, 3 d, 4 d, 7 d and 11 d aged embryos after 48 h imbibition (Fig 3).

The transcript abundance of alternative NADH dehydrogenases (*NDA1*, *NDA2*, *NDB1*, *NDB2* and *NDC1*), *NDA1* and *NDC1* showed a steady increase along with prolonged ageing treatment. In contrast, *NDA2* transcripts reached a minimum at 11 d. *NDB1* and *NDB2* also showed similar results, reaching a maximum at 7 d. In addition, with prolonged ageing treatment, the transcript abundance of the alternative oxidases (*AOX1a* and *AOX1c*) showed similar profiles, reaching a maximum at 7 d and 4 d, respectively. The transcript of *AOX1b* could not be reliably detected (data not shown). The transcript levels of two isoforms of the uncoupled protein (*UCP1* and *2*) showed that ageing treatment significantly increased their message levels. The transcript abundance of the COX (*COX5b*, *COX5c* and *COX6b1* and *2*), *COX5b* and *COX5c* message levels steadily reduced with prolonged ageing treatment. The transcript levels of *COX6b1* and *2* showed no significant changes. In general, this data indicated that the transcripts of the bypass components of the ETC were significantly induced during the CN of seed ageing, whereas the transcripts of the COX pathway are decreased.

### Respiratory activity and protein composition of isolated mitochondria

**Fig 4:**
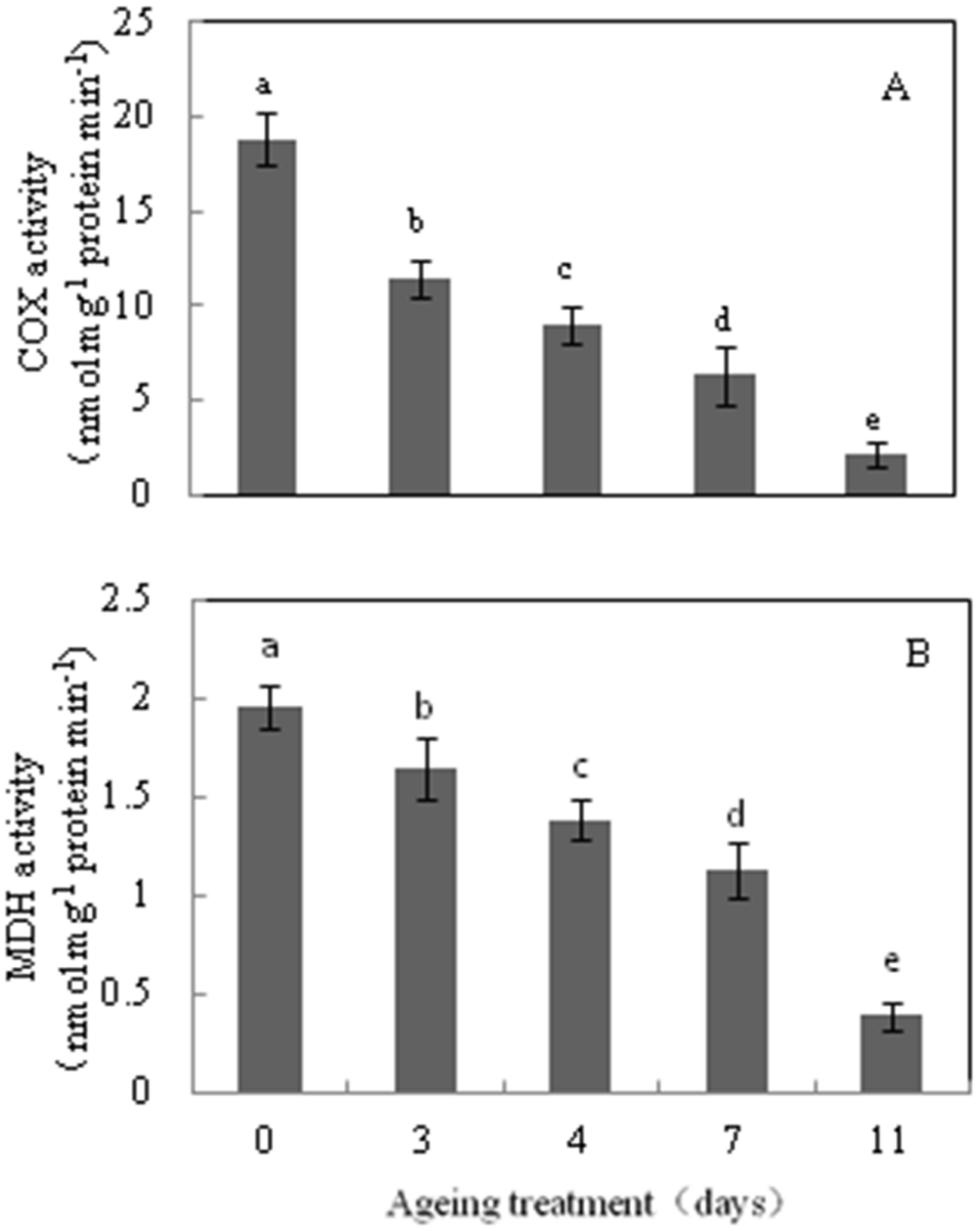
Cytochrome *c* oxidase (COX, A) and malate dehydrogenase (MDH, B) activity in crude mitochondria from 0 d, 3 d, 4 d, 7 d, and 11 d aged seeds. Data represents mean ± standard deviation of 3 independent experiments. All treatment significantly differed from the control (***p***< 0.05, one-way ANOVA, n = 3).

To assess mitochondrial functionality directly during the CN of seed ageing, oxygen consumption and the activities of COX and MDH were measured by crude and purified mitochondrial samples from aged seeds after 48 h imbibition. While it was difficult to find mitochondria by morphological examination in 7 d aged rice seeds, mitochondria were purified by a Percoll step gradient procedure from 0 d, 3 d, 4 d, 7 d and 11 d aged seeds after 48 h imbibition. However, purified mitochondria could not be obtained at the 25%:40% Percoll interface from 7 d and 11 d aged seeds. Yet, compared to the control, low COX and MDH activity and oxygen consumption were detected in crude mitochondria from 7 d and 11 d aged seeds (Fig 4 and Table 1). These results indicate that mitochondrial integrity is severely inhibited during imbibition when seed viability is below the CN.

**Table 1:** O_2_ consumption by crude mitochondria from aged seeds. O_2_ consumption rates are presented as means ± SD (n = 3).

Substrate	O_2_consumptionrate (*nmol·O*_*2*_*·min*^*-1*^*·mg*^*-1*^ *protein*)				
	0 d	3 d	4 d	7 d	11 d
**NADH**	75 ± 4	36 ±3	24 ± 2	10 ± 1	6 ± 0.4
**NADH+ADP**	140 ± 3	87 ± 4	60 ± 5	19 ± 2	14 ± 2
**Succinate**	38 ± 1	25 ± 5	13 ± 1	5 ± 1	3 ± 0.5
**Succinate+ADP**	54 ± 1	41 ±3	32 ± 3	11 ± 2	9 ± 1

**Fig 5:**
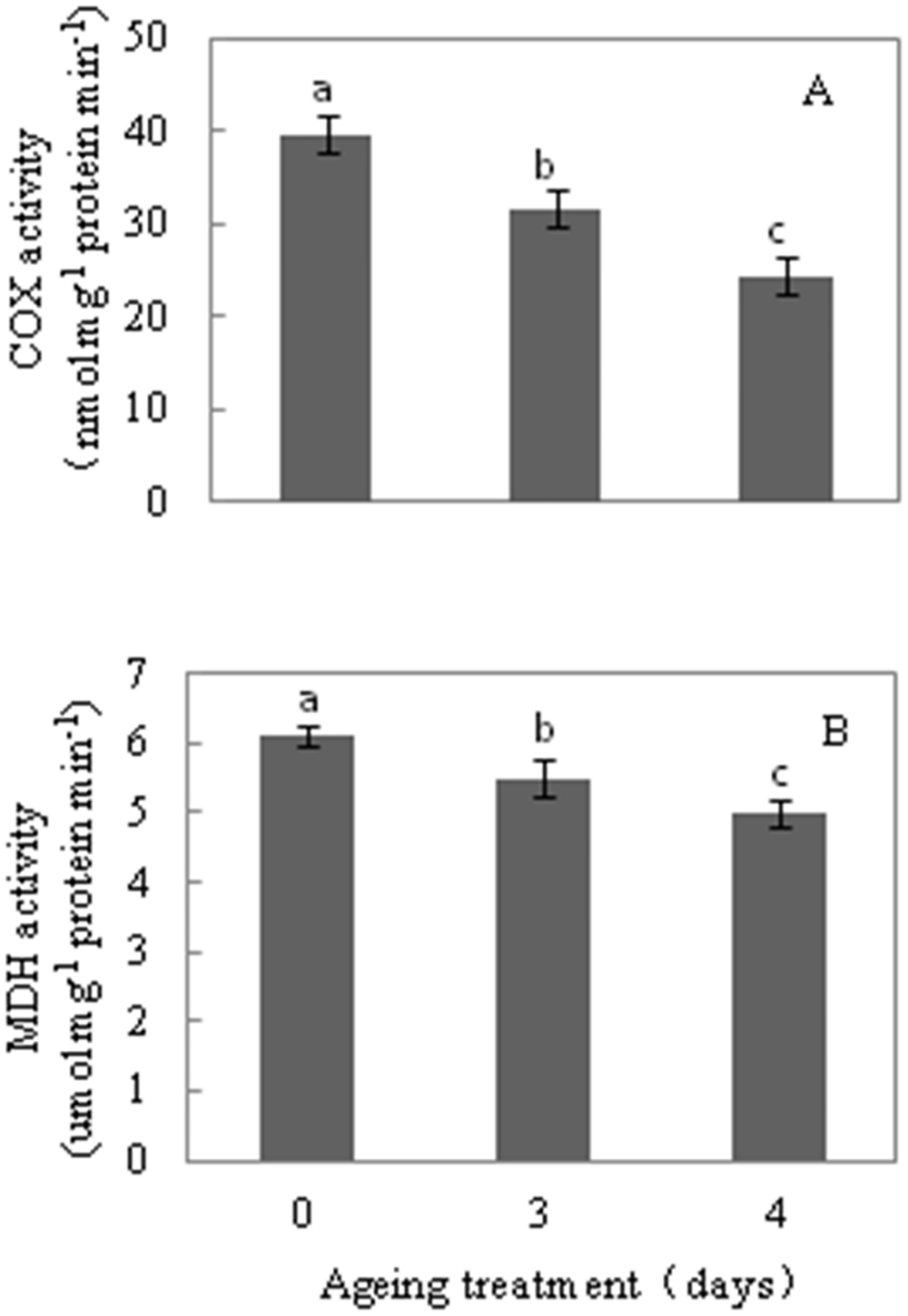
Cytochrome *c* oxidase (COX, A) and malate dehydrogenase (MDH, B) activity in purified mitochondria from 0 d, 3 d, and 4 d aged seeds. Data represent the mean ± standard deviation of 3 independent experiments. All treatment significantly differed from the control (***p***< 0.05, one-way ANOVA, n = 3).

To obtain a better understanding about the change to mitochondrial metabolism in the CN, mitochondria samples were purified by a Percoll gradient step procedure from 0 d, 3 d and 4 d aged seeds. Compared to the control, mitochondria from 3 d aged seeds showed a 24% and 10% decrease in COX and MDH activity, respectively (Fig 5), and a 44.5% and 25% decrease in NADH and succinate dependent O_2_ consumption, respectively (Table 2). Moreover, by 4 d of ageing treatment, mitochondria exhibited a 44% and 19% decrease in COX and MDH activity (Fig 5), whereas the capacity of NADH and succinate dependent O_2_ consumption decreased by 57% and 40% (Table 2), respectively. These results indicate that mitochondrial ETC is compromised in the CN, either via biogenesis, stability or a combination of both.

**Table 2:** O_2_ consumption by purified mitochondria from 0 d, 3 d, and 4 d aged seeds. O_2_ consumption rates are presented as means ± SD (n = 3).

Substrate	O_2_consumptionrate (*nmol·O*_*2*_*·min*^*-1*^*·mg*^*-1*^ *protein*)		
	0 d	3 d	4 d
**NADH**	95 ± 3	48 ± 4	35 ± 1
**NADH+ADP**	184 ± 9	102 ± 5	79 ± 6
**Succinate**	52 ± 3	37 ± 4	32 ± 3
**Succinate+ADP**	110 ± 5	82 ± 7	66 ± 2

**Fig 6:**
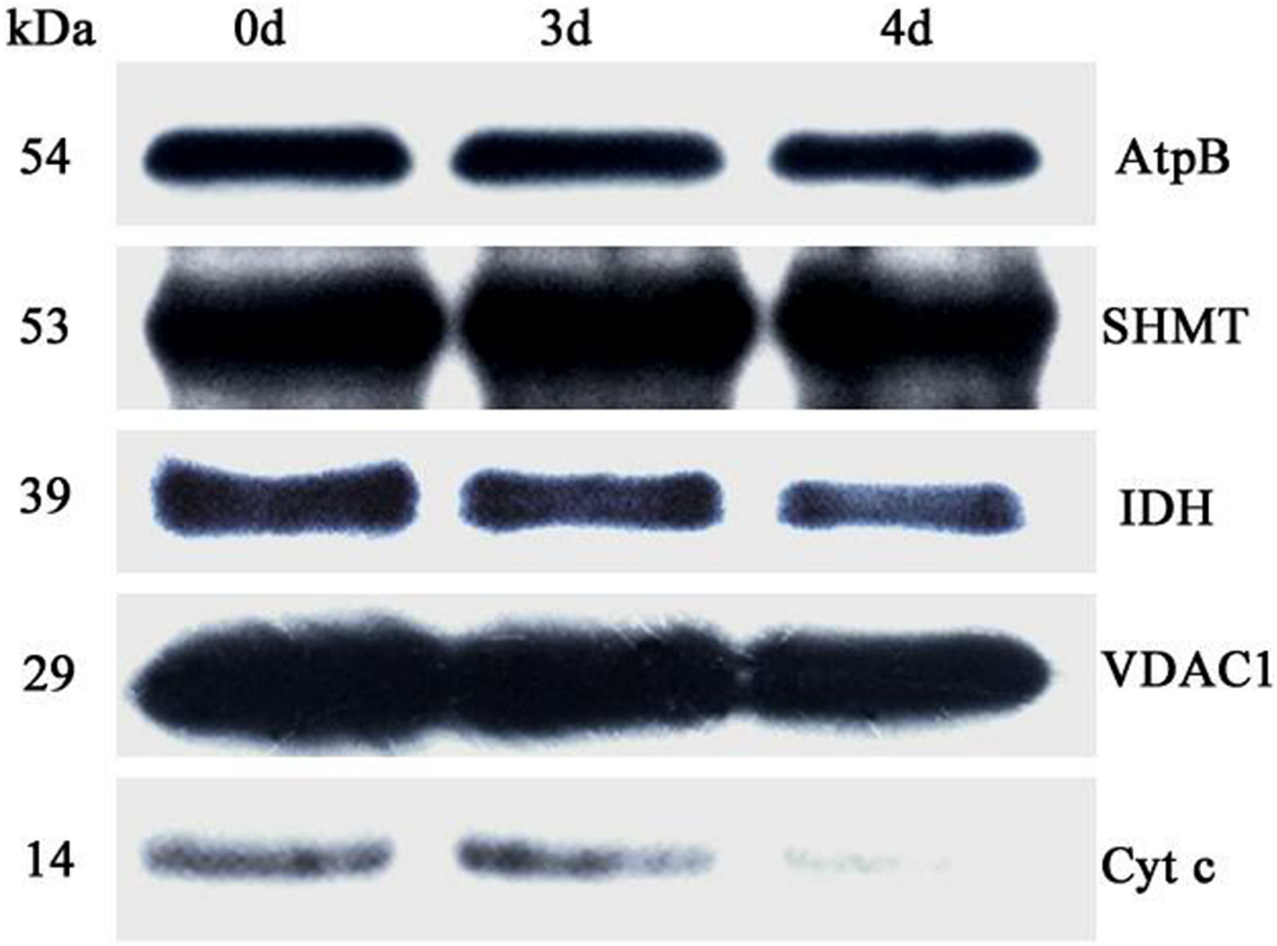
Abundance of a variety of known mitochondrial proteins by the immunoblot analysis of mitochondria isolated from 0 d, 3 d, and 4 d aged rice embryos after 48 h imbibition. Total 10 μg protein was separated by SDS gel electrophoresis and blotted to supported polyvinylidene difluoride, then probed with antibodies against beta subunit of ATP synthase (AtpB), serine hydroxymethyltransferase (SHMT), isocitrate dehydrogenase (IDH), voltage dependent anion channel 1 (VDAC1) and cytochrome *c* (Cyt *c*).

**Fig 7:**
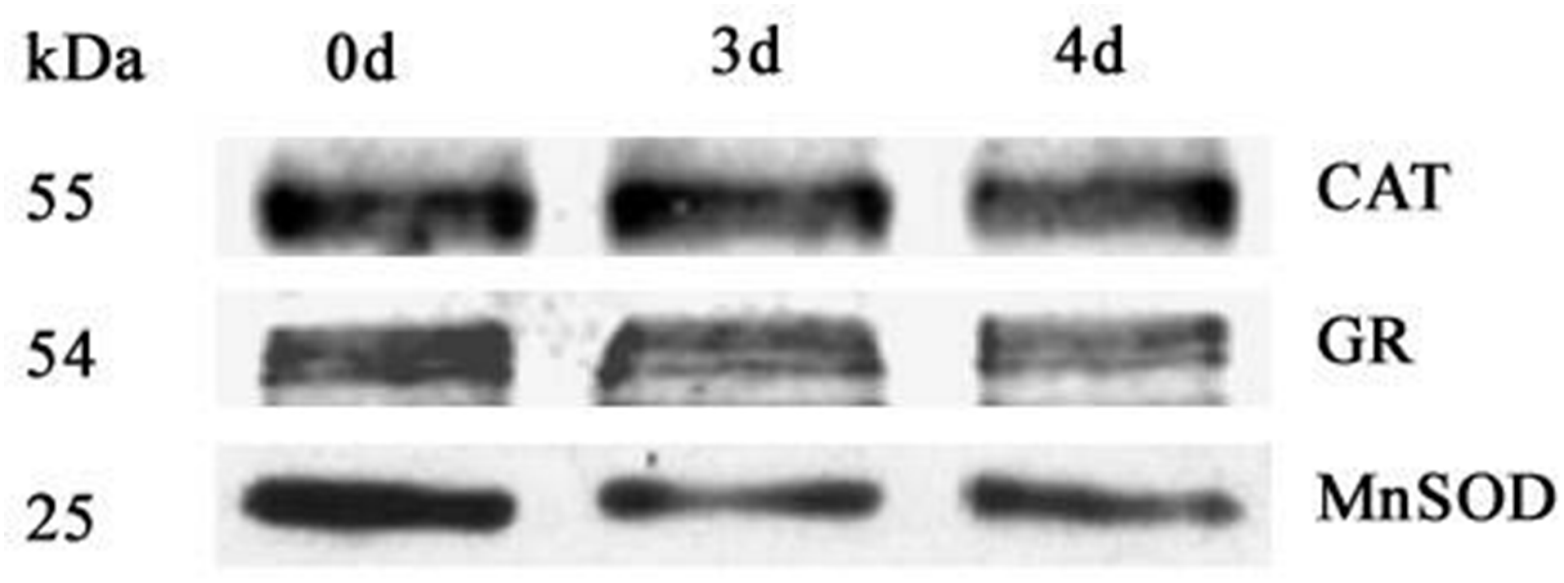
Western blot analysis of antioxidant enzymes in purified mitochondria from 0 d, 3 d, and 4 d aged seeds. Total 10 μg protein was separated by SDS gel electrophoresis and blotted to supported polyvinylidene difluoride, then probed with antibodies against catalase (CAT), glutathione reductase (GR) and manganese superoxide dismutase (MnSOD).

To further investigate changes in mitochondrial protein abundance in the CN, immunodetection was carried out in purified mitochondria from 0 d, 3 d, and 4 d aged seeds (Fig 6). Compared to the control, the beta subunit of ATP synthase (ATPase) (AtpB) and isocitrate dehydrogenase (IDH) only slightly decreased in mitochondria from 3 d and 4 d aged seed, whereas voltage dependent anion channel 1 (VDAC1) and cyt *c* dramatically decreased. However, the abundance of serine hydroxymethyltransferase (SHMT) did not change. We also analyzed mitochondrial antioxidative enzymes with antibodies for manganese superoxide dismutase (MnSOD), glutathione reductase (GR), ascorbate peroxidase (APX) and catalase (CAT). As shown in Fig 7, compared to the control, the mitochondrial GR and CAT slightly decreased in 3 d and 4 d aged seeds, with a significant reduction occurring in MnSOD. APX could not be detected. Overall, the results indicate that the anti-oxidative capacity of mitochondria decreases during imbibition in CN.

### Analysis of the proteome

**Fig 8:**
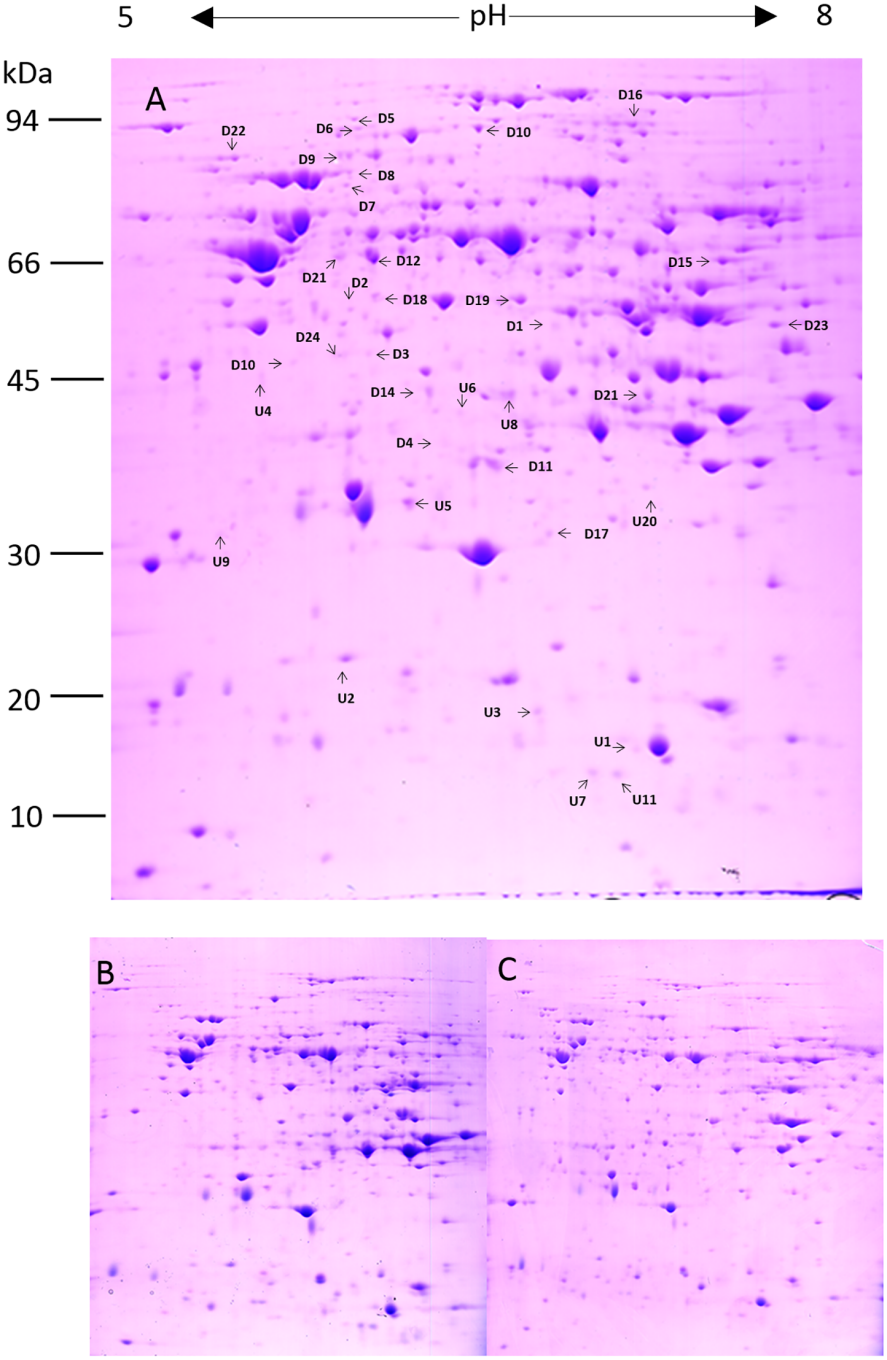
Representative IEF/SDS–PAGE separation gels of mitochondrial proteins from 0 d (A), 3 d (B) and 4 d (C) aged rice seeds after 48 h imbibition. The proteins were separated by first-dimensional pH 5–8 linear IPG strips and 12% SDS-PAGE gels in the second dimension. The proteins were numbered in a preparative 2D electrophoresis gel and excised for MS/MS analysis, corresponding to the proteins in Table 3. Number on the left represents the apparent molecular mass. Number above the gels represents the pI of separated protein spot.

To further survey the profiles of mitochondrial protein during the CN, proteins were extracted from 0 d, 3 d and 4 d aged rice embryo mitochondrial samples, and were subjected to two-dimensional electrophoresis (Fig 8, S1 Fig). First dimension separation utilized isoelectric focusing (IEF) on an immobilized pH linear gradient of pH 5–8. The second dimension was separated by 12% SDS–PAGE. Three independent experiments were carried out to obtain reliable spot information. More than 300 protein spots were detected in the individual gels from 0 d, 3 d and 4 d aged treatments, after excluding very faint spots and spots with undefined shapes and areas (Fig 8). Protein spots for which the relative abundance changed by at least 2-fold were designated as spots of interest, and were excised and identified by mass spectrometry. A total of 35 differentially expressed proteins were detected, including 24 down-regulated and 11 up-regulated proteins. Using the NCBInr database, 29 of the 35 spots were identified with MOWSE scores that were significantly higher than the threshold (*p*< 0.05). These spots included 19 down-regulated proteins and 10 up-regulated proteins (Table 3). The subcellular location of the identified proteins was predicted using plant tool of iPSORT. Most of the identified proteins were designated as being located in mitochondria. Seven proteins were predicted to be located in both the mitochondrion and plastid, and so may be distributed in both cellular organs. However, the presence of spot D2 (vacuolar ATPase subunit C), D14 (glucose and ribitol dehydrogenase homolog), and D16 (1, 4-alpha-glucan branching enzyme), in addition to spots U3, U4, U5, U7, and U8 (class I heat shock protein, Glutelin type-A1, Putative branched-chain alpha-keto acid decarboxylase E1 beta subunit, Glutelin type-A1 and Guanine nucleotide-binding protein subunit beta-like protein A), may indicate contamination by other cellular organs (Fig 8). These cytosolic proteins may have a functional association between the cytosol and mitochondria. Table 3 shows a significant decrease in mitochondrial protein levels. Of these down-regulated proteins, D6 (phosphate dikinase 1), D7 (phospoglycerate mutase), D12 (dihydrolipoyllysine residue acetyltransferase), D15 (fumarate hydratase 1), and D19 (methylcrotonoyl CoA carboxylase subunit) are involved in carbon metabolism. D4 (ascorbate peroxidase), D5 (heat shock protein), D11 (membrane pore protein), D10 (ATP dependent zinc metalloprotease FTSH 8), and D17 (MnSOD) are involved in oxidative stress responses. Two protein spots are nitrogen metabolism related enzymes, including D1 (phosphoglycerate dehydrogenase-like protein) and D3 (branched chain amino acid aminotransferase 5). Two protein spots (D13 and D18) were identified as elongation factor Tu, which may play a crucial role in mitochondrial protein biosynthesis. However, they might have different predicted locations, with D18 possibly being distributed in both mitochondria and plastids, while D13 may only be distributed in mitochondria. D8 (uncharacterized protein) has a pentatricopeptide repeat domain, which belongs to the pentatricopeptide family. The other identified protein showed the greatest increase in abundance. U1 (NADH:ubiquinone oxidoreductase 29 kDa subunit) is a subunit of the mitochondrial ETC NADH dehydrogenase. Increases in the levels of heat shock proteins (U2 and U9) were observed. Although U3 is also a heat shock protein, it was located in the cytosol. U6 (pyruvate dehydrogenase E1 component subunit beta 2) is a tricarboxylic acid (TCA) cycle related enzyme. Several spots (D20, D21, D22, D23, D24 and U11) did not fit with the database and require further investigation for identification. Overall, these increases and decreases of various mitochondrial proteins indicated that mitochondrial function was altered during the CN.

**Table 3:** Identification of rice embryo mitochondrial proteins from 0 d, 3 d, and 4 d aged seeds using MS/MS peptide spectra matched to iPSORT and NCBI entries. Compared to the control, fold changes were represented by a cut off following a 2-fold increase (Up-regulated) or decrease (Down-regulated).

Spot	Protein name	Accession	Score	Number of masses matched	Predicted location
**Down-regulated**					
D1	phosphoglycerate dehydrogenase-like protein	BAD31969.1	521	6	M
D2	vacuolar ATP synthase subunit C	AAO72561.1	170	3	other
D3	branched-chain-amino-acid aminotransferase 5	ABF94786.1	320	4	M
D4	ascorbate peroxidase	NP_001066305.1	419	5	M and P
D5	chaperone protein	CAE05148.2	446	10	M and P
D6	phosphate dikinase 1	NP_001055507.1	191	9	M
D7	phospoglycerate mutase	NP_001044625.1	324	4	M and P
D8	uncharacterized protein	EEE64481.1	136	3	M and P
D9	mitochondrial Rho GTPase	NP_001051665.1	428	9	M
D10	ATP-dependent zinc metalloprotease FTSH 8	NP_001055745.1	493	9	M
D11	membrane pore protein	BAB20636.1	180	2	M
D12	Dihydrolipoyllysine residue acetyltransferase	EEC72305.1	526	7	M
D13	translational elongation factor Tu	NP_001051912.1	261	4	M
D14	Glucose and ribitol dehydrogenase homolog	Q75KH3.2	301	7	other
D15	fumarate hydratase 1	ABF95833.1	834	9	M
D16	1,4-alpha-glucan branching enzyme	BAA01616.1	366	6	other
D17	manganese superoxide dismutase	AAA57130.1	184	2	M
D18	translational elongation factor Tu	AAF15312.1	198	3	M and P
D19	Methylcrotonoyl-CoA carboxylase subunit	NP_001067226.1	518	7	M
D20	unknown				
D21	unknown				
D22	unknown				
D23	unknown				
D24	unknown				
**Up-regulated**					
U1	NADH:ubiquinone oxidoreductase 29 kDa subunit	BAD23190.1	356	5	M
U2	heat shock protein	NP_001048175.1	382	6	M
U3	class I heat shock protein	NP_001041954.1	302	3	other
U4	Glutelin type-A 1	CAA29149.1	110	3	other
U5	Putative branched-chain alpha-keto acid decarboxylase E1 beta subunit	NP_001058989.1	645	9	other
U6	Pyruvate dehydrogenase E1 component subunit beta-2	NP_001063627.1	476	9	M
U7	Glutelin type-A 1	CAA29149.1	159	6	other
U8	Guanine nucleotide-binding protein subunit beta-like protein A	NP_001043910.1	340	5	other
U9	heat shock protein	BAD35228.1	652	8	M and P
U10	Glutathione-S-transferase–like protein	NP_001052302.1	305	5	M
U11	unknown				

## Discussion

It is recognized that seed vigor may be affected by mitochondrial activity. However, few studies have investigated the mechanism of seed ageing in relation to perturbed mitochondrial metabolism; particularly the changes in mitochondrial metabolism during at the CN. While inhibition of mitochondrial biogenesis from promitochondria during imbibition may be lethal, the loss in production of ATP and its intermediates has been shown to be associated with soybean seed ageing [30]. In this study, rice seed vigor rapidly declined from 97% to 92%, 84%, 61%, 22% and 8% after artificial ageing treatment for 3 d, 4 d, 7 d, 11 d and 14 d at 40°C and 75% RH, respectively (Fig 1). These seeds were used to study the changes in mitochondrial metabolism during the CN of seed ageing.

Previous studies have detailed the ultrastructural and physiological aspects of mitochondrial biogenesis from promitochondria during seed imbibition under normal germination conditions [22, 23, 25]. Compared with the embryos from 0 d aged rice seeds, aged seeds were subject to significant alteration in ultrastructure. Changes included the loss of cristae and the membrane in mitochondria, an irregular cytoplasmic layer, and an undeveloped nuclear structure (Fig 2). The transmission electron microscopy results showed that mitochondrial enzyme activity and oxygen consumption were paralleled by damage to the mitochondrial ultrastructure in aged seeds (Fig 4 and Table 1). Our results are consistent with the finding reported in aged soybean, showing that damage to the mitochondrial ultrastructure may directly impair mitochondrial function [27]. Several observations are worth noting about the responses of mitochondrial metabolism to the 7 d ageing treatment: i) the ultrastructure was that of promitochondria; ii) enzyme activity was extremely low; and iii) the respiratory capacity was limited. These responses are consistent with the concept that mitochondria in aged cells have restricted activity and cannot sufficiently produce ATP and its intermediates to maintain seed germination when seed viability is in the CN, which leads to a rapid decrease in the germination percentage.

In addition, oxygen uptake measurements indicated that NADH and succinate-dependent respiratory activity are reduced in aged seeds (Tables 1 and 2), due to their restricted respiratory pathways. Plant mitochondria contain multiple energy dissipating components in the ETC, including the cytochrome pathway (COX) and alternative pathways (AOX, ND, and UCP) [35, 39, 40]. The transcript abundance of genes encoding proteins of the mitochondrial ETC proteins was significantly changed in aged embryos (Fig 3). The expression of nuclear genes encoding mitochondrial proteins is orchestrated by mitochondria retrograde regulation, which is triggered by mitochondrial dysfunction, such as the disruption of electron transport and the accumulation of ROS [41]. Transcript abundance of *COX* genes steadily declined with prolonged ageing treatment (Fig 3). In contrast, the transcript abundance of *AOX*, *UCP* and *ND* gene families increased. This result was consistent with a number studies that reported *AOX*, *UCP* and *ND* gene families display co-expression under stress in *Arabidopsis* [42–44]. Supporting previous studies, we showed that COX and MDH activity significantly decreased as seeds aged (Figs 4 and 5). These observations indicate that the artificial ageing treatment interfered with the classic mitochondrial ETC, and induced the mitochondrial bypass of ETC. Of note, compared with 4 d aged embryos, the *AOX*, *UCP* and *ND* gene family transcripts were lower or remained unchanged in the 7 d and 11 d aged embryos, except for *AOX1a* and*NDC1*. In contrast, the transcript abundance of the *COX* gene families was extremely low. Thus, these results indicate that seeds might regulate the transcript expression of cytochrome and alternative pathways. When seed viability was in the CN, a slight decrease in the capacity of the cytochrome pathway may result and alternative pathway may be triggered. When seed viability was lower than the CN, both pathways were inhibited. However, further evidences about mitochondria retrograde regulation in plants are required.

The reverse change in abundance of cytochrome and alternative pathways during seed ageing CN (Fig 3), was accompanied by changes in mitochondrial proteins. Given the large magnitude of changes to mitochondrial proteins observed in 0 d, 3 d and 4 d aged rice seeds, both Western blotting and proteomic analysis of the purified mitochondria were carried out (Figs 6–8 and Table 3). These results provided further evidence that mitochondrial proteins are significantly decreased in aged seeds after imbibition. The protein levels for carbon and nitrogen metabolism decreased, indicating a decrease in the activities of the matrix. The transmission electron microscopy results showed that the matrix decreased in aged seeds (Fig 2). These results were similar to those obtained by Sweetlove et al [45] and Taylor et al [5], in which the matrix enzymes were degraded in Arabidopsis and pea mitochondria under stress. In contrast, increases in matrix components were observed in rice and maize mitochondria after seed imbibitions [22, 23]. This phenomenon may reveal that the assembly of matrix components is inhibited during imbibition in aged seeds. Not surprisingly, IDH which is a classic enzyme component of the TCA cycle in the matrix [46], decreased in aged seeds (Fig 6). These results indicated that TCA cycle activity decreased in aged seeds. The TCA cycle is critical for mitochondrial metabolism. A decrease in TCA cycle activity may cause ROS accumulation, further increasing oxidative damage [47]. In the aged rice seeds, the accumulation of H_2_O_2_ and superoxide were consistent with the down-regulation of antioxidant enzyme expression, along with their protein translation [48]. Mitochondria are the major site for the production and scavenging of ROS in response to stress [49]. In particular, MnSOD, which is involved in scavenging superoxide radicals produced in the mitochondria [49], was reduced far more than CAT and GR in the mitochondria from 3 d and 4 d aged rice seeds (Fig 7). Interestingly, MnSOD (D17) and APX (D4) decreased in the 2 D gels. This result indicated that mitochondrial redox homeostasis was disturbed in aged seeds. This response was consistent with previous findings that mitochondrial ascorbate and glutathione activity are reduced in aged soybean seeds [28].

Mitochondrial VDAC1 is an outer membrane channel that allows metabolite transport across the mitochondria and cytosol, including pro-apoptotic proteins [50]. In aged seeds, there was a slight decrease in VDAC1 (Fig 6). Our results are consistent with previous reports showing that the level of VDAC deceased under abiotic stress, whereas VDAC overexpression enhanced tolerance [51–53]. The decrease in VDAC1 levels might cause a decrease in cyt *c*. As expected, cyt *c* severely decreased in aged seeds (Fig 6). The release of cyt *c* might be a signal to induce the cell death program [54]. Like VDAC1, the membrane pore protein (D 11) decreased in aged seeds (Fig 8). According to its domain, the membrane pore protein is that like a member of the mitochondrial translocase inner membrane (TIM) family. These results were consistent with the transmission electron microscopy results, indicating that the mitochondrial membrane system was ruptured (Fig 2), causing the loss of mitochondrial membrane structure and function in aged seeds.

Oxidative stress suppresses *ATPB* gene expression [45]. *ATPB* levels were slightly decreased in aged seeds (Fig 6), representing the F_o_ sector of membrane-bound ATPase. Like cyt *c*, ATPB can directly or indirectly promote cell death under stress [55, 56]. Interestingly, vacuolar ATPase subunit C (D2, AtpC), which is the F_1_ sector of ATPase, decreased in aged seed (Fig 8). Mitochondrial ATPase is formed from F_1_ and F_o_ components [57, 58]. Stress treatments can suppress the expression of F_o_F_1_ ATPase and degrade the supercomplex [45]. This result indicated that the assembly of mitochondrial ATPase was inhibited in aged seed, causing ATP production to decrease. This result is consistent with the decrease in oxygen consumption (Table 2).

## Conclusions

The present study indicates that the respiratory capacity of aged seeds is restricted at the CN. This response is related to the morphologically impaired induction of the alternative pathway and the inhibition of the cytochrome pathway, leading to decreased protein production. These events might cause the production of ATP and its intermediates to be inhibited, along with signals from mitochondria, leading to a decrease in cyt *c* and the accumulation of ROS, which may induce oxidative damage. The mitochondrial protein levels related to carbon and nitrogen metabolism, ATP synthase (ATPase) complex, tricarboxylic acid cycle (TCA) cycle are down-regulated which may cause the mitochondrial dysfunction. However, the way in which these events are regulated requires further research.

## Supporting Information

S1 Fig All replicate Gel images. Representative IEF/SDS–PAGE separation gels of mitochondrial proteins. (RAR)

S1 Table List of genes used in real-time PCR. (DOCX)
