# Supplementary material for: Comprehensive Mitochondrial Metabolic Shift during the Critical Node of Seed Ageing in Rice
Source: PLoS One. 2016 Apr 28;11(4):e0148013. doi: 10.1371/journal.pone.0148013 (PMC4849721; doi:10.1371/journal.pone.0148013)
Supplement: S1 Table — (DOCX) [file pone.0148013.s002.docx]

## S1 table. List of genes used in real-time PCR.

| Gene name | Accession/Locus | Gene description |
| --- | --- | --- |
| *AOX1a* | AK064040 | Alternative oxidase 1a |
| *AOX1c* | AP004778.3 | Alternative oxidase 1c |
| *COX5b* | D85381 | Cytochrome c oxidase subunit 5b |
| *COX5c* | AB027123 | Cytochrome c oxidase subunit 5c |
| *COX6b1* | NC_003070 | Cytochrome c oxidase subunit 6B1 |
| *COX6b2* | AB047976 | Cytochrome c oxidase subunit 6B2 |
| *NDA1* | LOC_Os07g37730 | type II NAD(P)H dehydrogenases A1 |
| *NDA2* | LOC_Os01g61410 | type II NAD(P)H dehydrogenases A2 |
| *NDB1* | LOC_Os06g47000 | type II NAD(P)H dehydrogenases B1 |
| *NDB2* | LOC_Os05g26660 | type II NAD(P)H dehydrogenases B2 |
| *NDC1* | LOC_Os06g11140 | type II NAD(P)H dehydrogenases C1 |
| *UCP1* | AB049997 | uncoupling protein 1 |
| *UCP2* | AB049998 | uncoupling protein 2 |
| *UBQ5* | AK062354 | Ubiquitin 5 |
